# Moderate exercise mobilizes Prg4+ cells in the superficial zone of articular cartilage and maintains cartilage homeostasis

**DOI:** 10.1016/j.bj.2026.100989

**Published:** 2026-05-05

**Authors:** Peichu Tian, Yaojun Suo, Li Guo, Pengcui Li, Jiazhu Chen, Jiawei Zhang, Yongsheng Ma, Xuanping Zhang, Chunfang Wang, Xiaochun Wei

**Affiliations:** a College of Basic Medicine, Shanxi Medical University, Taiyuan, Shanxi, China; b Shanxi Key Laboratory of Bone and Soft Tissue Injury Repair, Department of Orthopedics, Second Hospital of Shanxi Medical University, Taiyuan, Shanxi, China; c Department of Anesthesia, the Children's Hospital, Zhejiang University School of Medicine, National Clinical Research Center for Child Health, Hangzhou, Zhejiang, China; d Shanxi Pharmaceutical Vocational College, Taiyuan, Shanxi, China

**Keywords:** Superficial zone, Prg4, Exercise, Osteoarthritis, Cre-loxP

## Abstract

Different exercise intensities exert distinct effects on the knee joint. Overuse of the knee joint may lead to the early onset of osteoarthritis (OA), which is usually associated with injury to the superficial zone (SFZ) of articular cartilage. Proteoglycan 4 (PRG4), a representative marker of the SFZ, plays a pivotal role in articular cartilage lubrication and is mainly secreted by SFZ cells.In this study, Cre-dependent Prg4 tracing mice and wild-type mice were used, and all mice were subjected to treadmill running to induce forced movement. Additionally, the proliferation and pluripotency of SFZ cells were investigated, with relevant markers including Pcna, CD34, and others.As expected, the red fluorescence of Prg4 was significantly upregulated under moderate exercise but downregulated under excessive exercise. However, no significant changes were observed in proliferation- and stem cell-related markers, except for CD34 in the excessive exercise group. Furthermore, several anabolism and catabolism markers, such as Collagen 2, Aggrecan, and Mmp13, did not show significant changes during the exercise process.In summary, Prg4 expression increased under moderate exercise and slightly decreased under excessive exercise, which could provide guidance for formulating appropriate exercise patterns. Moreover, immunohistochemical analysis revealed that Caspase-3, one of the apoptosis markers, exhibited the same expression tendency as Prg4 during exercise.Collectively, this study demonstrated the expression patterns of Prg4 and other related markers under different exercise intensities, confirming that moderate exercise enhances Prg4 expression and that Prg4 maintains a relatively high level even under excessive exercise. Furthermore, Prg4 may regulate Caspase-3 expression through a specific signaling pathway.

## 1 Introduction

Osteoarthritis (OA) is a degenerative and inflammatory joint disorder that predominantly affects the knee joints. It manifests as joint pain, swelling, and limited mobility. In its advanced stages, patients frequently require total joint arthroplasty, which places a substantial economic burden on healthcare systems and represents a major societal challenge globally. Current management strategies for OA are mainly centered on symptomatic alleviation via physiotherapy, exercise, and other conservative interventions, yet no curative therapies exist to effectively regenerate articular cartilage.

Emerging evidence indicates that exercise may exert protective effects against osteoarthritis. Liu et al. [1] reported that exercise can protect chondrocytes from inflammation and pyroptosis in OA, enhance the restoration of type II collagen, and reduce the expression of MMP13 and ADAMTS5. In a rat model, treadmill exercise was similarly shown to effectively alleviate OA progression by upregulating anti-inflammatory mediators and suppressing pro-inflammatory cytokine production [2]. Although the beneficial effects of physical exercise on OA symptoms are widely recognized, excessive mechanical loading can conversely elevate the risk of OA development. For example, intensive downhill running in mice triggered early-onset knee OA accompanied by a chronic pro-inflammatory state, which was associated with premature cartilage degeneration and subchondral bone lesions [3]. In addition, insufficient lubricin secretion has been linked to Caspase-3 activation and sustained inflammatory responses in cartilage, which may provide novel insights into the pathogenesis of post-traumatic osteoarthritis [4,5].

Articular cartilage (AC) is an avascular and aneural tissue with extremely limited self-repair capacity. The precise etiology and initial pathological site of osteoarthritis (OA) remain incompletely defined. Pathologically, OA is characterized by the progressive degeneration of AC, which is structurally stratified into distinct zones: the superficial zone (SFZ), middle zone, deep zone, tidemark, and calcified zone [6]. Accumulating evidence has identified the SFZ as a likely initiation site for OA, underscoring the importance of clarifying its function in disease onset. The SFZ consists of flattened, elongated chondrocytes aligned parallel to the cartilage surface [7], in contrast to the deeper cartilage zones that are abundant in proteoglycans and collagen fibers [8]. This layer serves multiple critical functions in preserving joint homeostasis. Early investigations demonstrated that moderate combined compressive and shear forces upregulated the expression of PRG4 and other related markers, with zone-specific responses observed across the superficial, middle, and deep regions of articular cartilage [9].

Proteoglycan 4 (PRG4), also known as lubricin, serves as a key molecular marker of the superficial zone [10,11]. Genetic models such as *Prg4-CreERT2;Ai14* mice allow for the specific tracing of PRG4-expressing cells, representing a powerful tool for investigating the biology of the SFZ [12]. Featuring mucin-like domains, PRG4 is indispensable for maintaining proper joint lubrication. During osteoarthritis progression, even after erosion of the superficial cartilage layer, a small number of PRG4-expressing cells can still be detected [13].Therapeutic strategies targeting PRG4 have shown considerable potential. Intra-articular administration of PRG4 has been shown to mitigate cartilage damage and inflammation in a porcine post-traumatic OA model [14], while PRG4-based gene therapy has yielded beneficial effects in murine OA models [15,16]. Furthermore, studies have revealed that PRG4 deficiency contributes to early-onset OA, often in association with SFZ damage, whereas its overexpression exerts protective effects against cartilage degeneration [17].

Cre recombinase-mediated lineage tracing has greatly advanced our understanding of the cellular origins of SFZ cells and the regulatory mechanisms underlying cartilage development and growth [18,19]. Recent studies have reignited interest in SFZ biology by proposing that this region may harbor a population of stem or progenitor cells. In contrast to chondrocytes in the deeper cartilage zones, SFZ cells express a panel of stem cell-related markers, including CD105, CD34, and Sox2 [20].Lineage tracing analyses of skeletal progenitors have revealed that osteophytes can arise from Pdgfrα-positive stem cells, with Sox9-expressing progenitors giving rise to hybrid skeletal cells and Prg4-positive progenitors contributing to the cartilage cap of osteophytes [21]. Another investigation identified Hoxa11 as a progenitor marker co-expressed with PDGFRα/CD51 and LepR [22], highlighting the heterogeneity of progenitor cell populations during cartilage development and supporting the notion that Prg4 may serve as a defining progenitor marker for SFZ cells.

Accordingly, the present study employed treadmill exercise combined with Cre reporter mouse models to investigate the effects of physical exercise on the migration and distribution of Prg4, along with other key markers including CD34, Pcna, Collagen II, and Aggrecan, in the superficial zone. We sought to elucidate the regulatory mechanisms by which exercise modulates the function of the SFZ during osteoarthritis development and progression.

## 2 Materials and methods

### 2.1 Mice

Sixteen-week-old C57BL/6 mice were randomly allocated to three groups: a 4-week exercise group (n = 9), an 8-week exercise group (n = 9), and a sedentary control group (n = 9), with both male and female animals included. Mice were housed under standard laboratory conditions (ambient temperature, 12-h light/dark cycle, maximum 7 mice per cage) with ad libitum access to food and water [23]. All experimental procedures were approved by the Ethics Committee of the Second Hospital of Shanxi Medical University (approval No. DW2025047). Prg4^CreERT2^(C57BL/6Smoc-Prg4em(Kozak-CreERT2-2A-EGFP-WPRE-pA)1Smoc) and Rosa26^LSL-tdTomato^ (B6.Cg-Gt(ROSA)26Sortm9(CAG-tdTomato)Hze/J) mice were purchased from Shanghai Model Organisms Center, Inc. Heterozygous Prg4-CreERT2; ROSA26-LSL-tdTomato mice were bred and genotyped for use in this study. Tamoxifen administration was used to activate Cre recombinase, which excises the STOP cassette and drives tdTomato red fluorescence specifically in Prg4-expressing cells for lineage tracing [24]. These reporter mice were incorporated into all three experimental groups, with 3 animals per group.

### 2.2 Tamoxifen injection

Tamoxifen (Sigma-Aldrich, T5648) was dissolved in corn oil (Solarbio, C7031) at a concentration of 10 mg/mL using a 60 °C water bath, then aliquoted and stored at 4 °C. Prior to intraperitoneal injection, the solution was prewarmed to 37 °C [25]. Tamoxifen was then administered (100 mg/kg body weight) via intraperitoneal injection for 5 consecutive days before initiation of the exercise protocol.

### 2.3 Treadmill exercise

The exercise protocol was performed according to a previously established method [26]. Running intensity was adjusted individually based on each animal's aerobic capacity and maximum velocity (MV), as determined by a pre-training performance test. All mice ran 3 days per week (Monday, Wednesday, Friday). The 4-week exercise group completed a total of 450 min of running on a level treadmill (0° inclination). The 8-week group ran for a total of 1080 min: running on a level surface for the first 4 weeks, followed by 4 weeks of downhill running at a −14° inclination [Fig. 1]. These two regimens represented moderate and excessive exercise, respectively. Accordingly, the three groups are hereafter abbreviated as MOD (moderate exercise), EXC (excessive exercise), and CON (control).

**Fig. 1:**
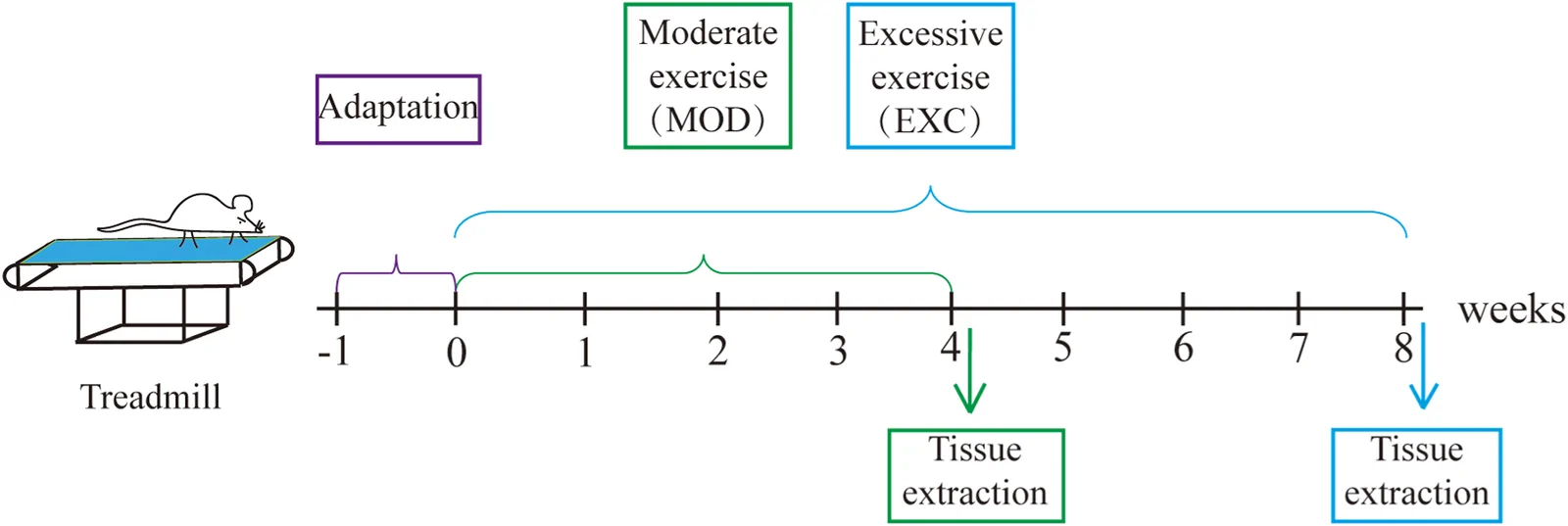
The exercise pattern of the experiment.

### 2.4 X-ray imaging

Knee joints (n = 6–8 per group) were subjected to X-ray imaging (Faxitron, USA) to evaluate joint morphological changes following exercise [27]. Anteroposterior and lateral views of the right hind limb were acquired. The anteroposterior scan was performed to fully visualize the knee joint space, whereas for lateral imaging, the knee joint was flexed to approximately 150°. Imaging exposure was performed using the instrument's default automatic parameters [28].

### 2.5 Fluorescence and imaging analysis

Cryosections were washed with phosphate-buffered saline (PBS), and cell nuclei were counterstained with DAPI solution (Solarbio, C0065, 10 μg/mL) for 20 min at room temperature. Following additional PBS rinses, sections were visualized under a confocal laser scanning microscope (Leica TCS SP8, Germany). Cryosections were washed with phosphate-buffered saline (PBS), and cell nuclei were counterstained with DAPI solution (Solarbio, C0065, 10 μg/mL) for 20 min at room temperature. Following additional PBS rinses, sections were visualized under a confocal laser scanning microscope (Leica TCS SP8, Germany).

### 2.6 Immunohistochemistry and histology

Knee joints were fixed in 4% paraformaldehyde overnight, decalcified in 0.5 M EDTA for 2 weeks, embedded in paraffin, and sectioned at 4–5 μm thickness (Leica RM2245, Germany). Sections were deparaffinized and rehydrated using standard protocols [29]. For frozen sections, tissues were cryoprotected in 30% sucrose, embedded in OCT compound, and serially sectioned at 7–8 μm. For immunohistochemistry, endogenous peroxidase activity was blocked with 3% H_2_O_2_, and nonspecific binding was minimized using normal goat serum, prior to incubation with primary antibodies at 4 °C overnight. Horseradish peroxidase (HRP)-conjugated secondary antibodies (Zsbio, Beijing, China) were then applied, followed by DAB chromogenic detection (Zsbio, Beijing, China). Sections were counterstained with hematoxylin (LEAGENE, Beijing, China) and digitized using a 3DHISTECH Panoramic MIDI scanner (3DHISTECH, Hungary) [29]. Detailed information on the primary antibodies used is listed in [Supplemental Table 1].

### 2.7 Safranin O staining and OA scoring

Deparaffinized and rehydrated sections were stained with 0.1% fast green (Solarbio, F8130) for 5 min, rinsed briefly in 1% acetic acid, and counterstained with 0.1% Safranin O (Sigma-Aldrich, S2255-25G) for 30 s. Following rinsing, sections were dehydrated, cleared, and mounted with coverslips [30].Cartilage lesions in the medial and lateral tibial plateaus as well as femoral condyles were scored by three blinded, independent researchers according to the OARSI grading system (0–6) [31].

### 2.8 HE staining and cartilage thickness

Additional sections were stained with hematoxylin and eosin (H&E). Cartilage thickness was measured in the medial and lateral tibial plateaus and femoral condyles using a 3DHISTECH quantitative analysis system (3DHISTECH, Hungary), and the average value was calculated for statistical analysis. Each histological group consisted of n = 6 mice.

### 2.9 Statistical methods

Data are presented as means with individual data points (n = 6–9). A p-value<0.05 was considered statistically significant. Statistical analyses were performed using Microsoft Excel 2019 and GraphPad Prism (version 10.3f). Group comparisons were conducted by one-way ANOVA, followed by pairwise post hoc testing using an unpaired two-tailed Student's *t*-test (assuming equal variances). GraphPad Prism was used to compute means, 95% confidence intervals, and mean differences, as well as to generate all graphical illustrations.

## 3 Results

### 3.1 Generation of Prg4-Reporter mice

We successfully generated Prg4-reporter mice [Fig. 2a]. No significant size difference was observed between wild-type and mutant strains [Fig. 2b]. Genotyping was confirmed by agarose gel electrophoresis [Fig. 2c]. Following tamoxifen injection, tdTomato red fluorescence was clearly visible in the knee joint cryosections, confirming the successful creation of the Prg4-reporter model [Fig. 2d].

**Fig. 2:**
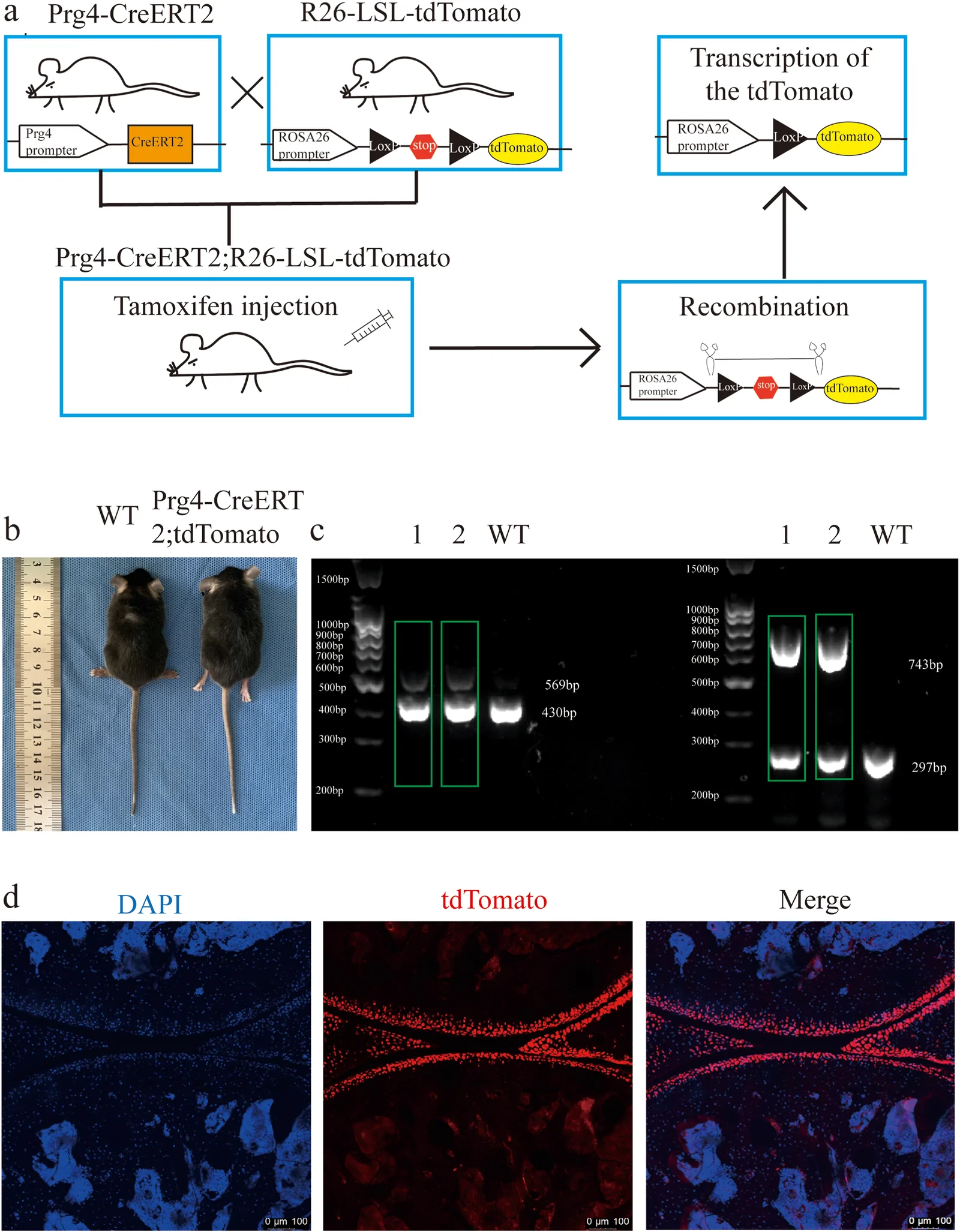
Prg4^+^ reporter mice(a):The schematic diagram of the production of Prg4+ cell reporter mice. Tamoxifen administration activates Cre recombinase, excising the STOP cassette and inducing tdTomato red fluorescence in Prg4-positive cells for lineage tracing (b): The macro photo of Prg4^+^ cell reporter mice and wild type mice, (c): The genotyping of Prg4^+^ cell reporter mice and wild type mice by using the agarose gel electrophoresis, (d): The histological image of knee joint of the Prg4+ cell reporter mice.

### 3.2 The normal histology

H&E and Safranin O/Fast Green (SOFG) staining [Fig. 3] revealed an intact cartilage surface with no superficial zone damage following moderate exercise. By contrast, the excessive exercise group displayed obvious surface abrasion and decreased glycosaminoglycan content, as reflected by weaker Safranin O staining intensity, indicative of detrimental effects. Nevertheless, neither OARSI scores nor quantitative cartilage thickness measurements showed statistically significant differences among groups, supporting the safety of moderate exercise and suggesting that the deleterious effects of excessive exercise were mild yet histologically discernible.

**Fig. 3:**
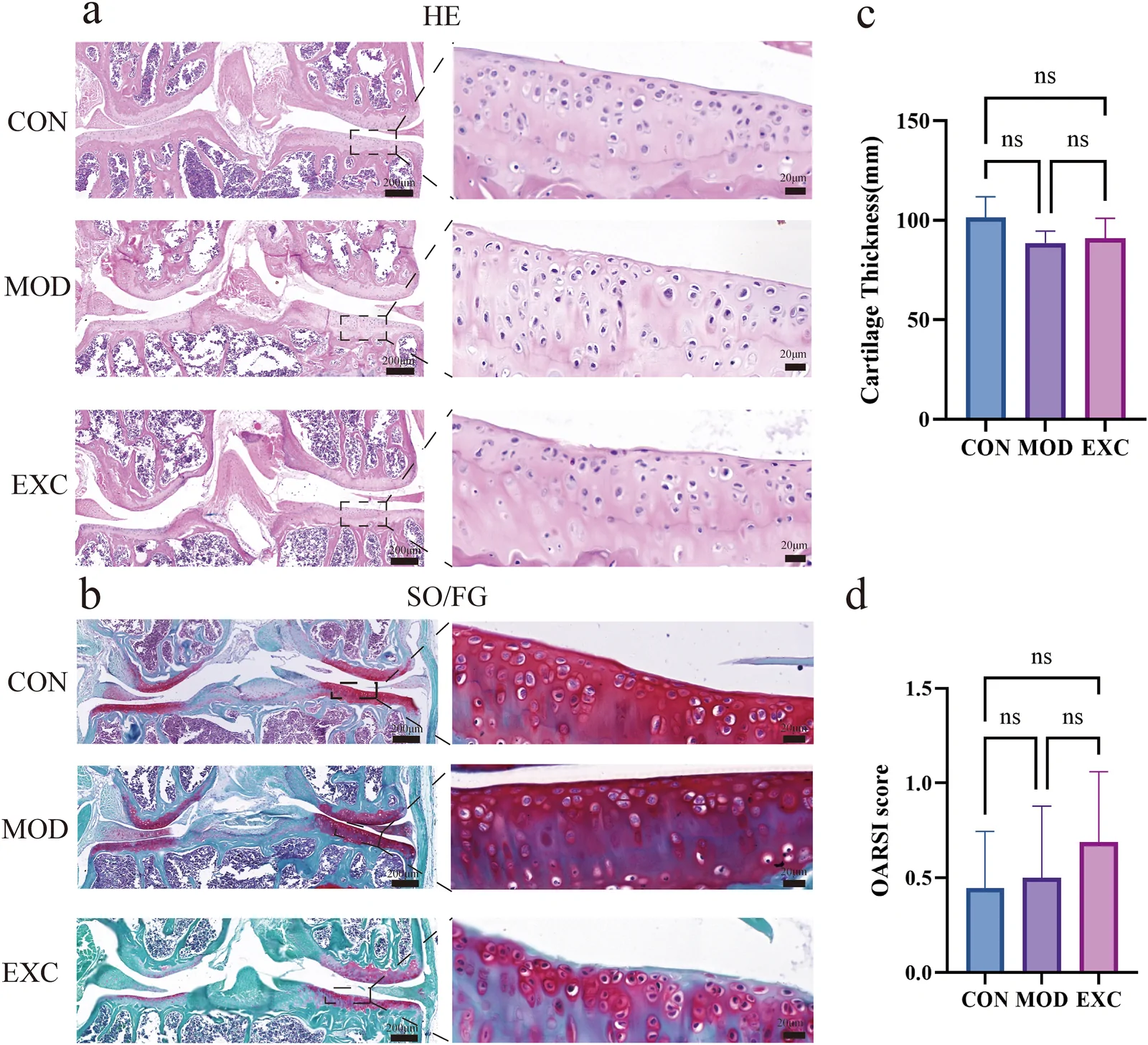
(a)(b) The representative image of HE staining and SOFG staining. (c)(d) The cartilage thickness using HE image, and the OARSI score using safranin O/Fast green staining. Data are expressed as the mean ± SD (n = 6 per group). Abbreviation: ns, no significance.

### 3.3 Prg4 is highly expressed when the exercise loading is moderate

Following moderate treadmill exercise, the fluorescence intensity of Prg4 (tdTomato) was markedly higher than that in the sedentary control group. Notably, Prg4+ cells were distributed in both the superficial and middle zones, in contrast to their predominant localization in the superficial zone in control mice [Fig. 4]. Conversely, excessive treadmill running resulted in reduced fluorescence intensity, and fewer Prg4+ cells were detected within articular cartilage compared with the moderate exercise group.Quantitative comparison of Prg4+ cell distribution between the superficial zone and the middle/deep zones further revealed an increased proportion of Prg4+ cells in the middle and deep regions, supporting the notion that exercise triggers the activation and migration of Prg4-expressing cells from the superficial zone toward deeper cartilage zones [Fig. 4d and e].

**Fig. 4:**
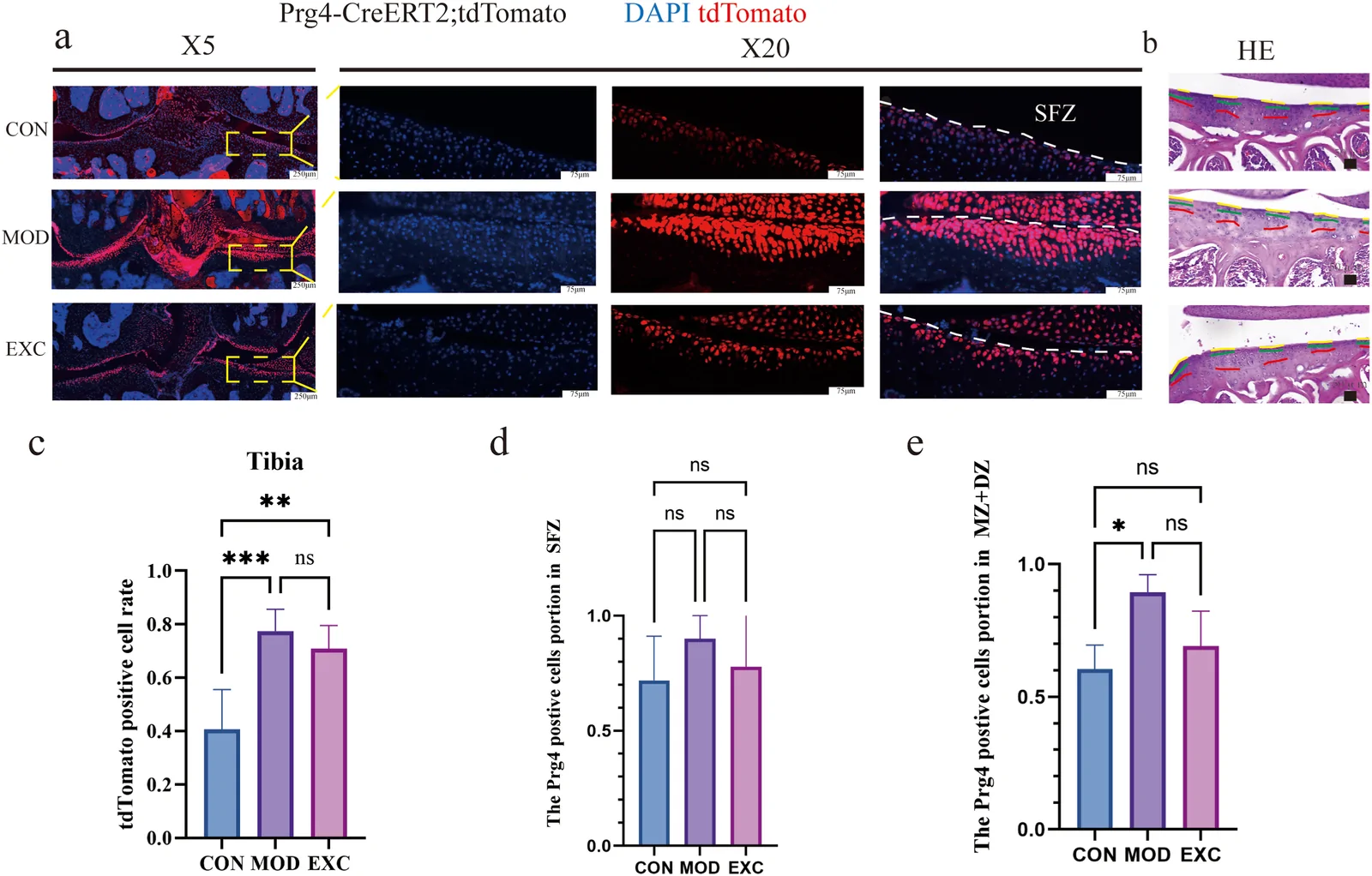
(a) The fluorescent images of different groups. (b) The related HE staining images in each group, the green line marks the boundary between the superficial zone (SFZ) and the middle + deep zone (MZ + DZ), and the red line represents the tidemark. (c) Quantification of tdTomato^+^ cells in the knee joints as shown above. (d) Quantification of Prg4^+^ cells portion in SFZ as shown above. (e) Quantification of Prg4^+^ cells in portion in MZ + DZ as shown above. Data are expressed as the mean ± SD (n = 6 mice per group, *p < 0.05 and **p < 0.01, ***p < 0.001). Abbreviation: ns, no significance.

Immunohistochemical staining for Prg4 protein confirmed consistent expression patterns [Fig. 5].Similar exercise-dependent alterations in Prg4 were observed in the meniscus and synovium of both lineage-tracing mice and wild-type littermates [Fig. S1–S3]. A recent study proposed that the synovium and intra-articular adipose tissue act as a functional unit in maintaining joint homeostasis, with Dpp4+ mesenchymal progenitors differentiating into Prg4+ synovial lining cells and adipocytes during skeletal development and osteoarthritis progression [32].

**Fig. 5:**
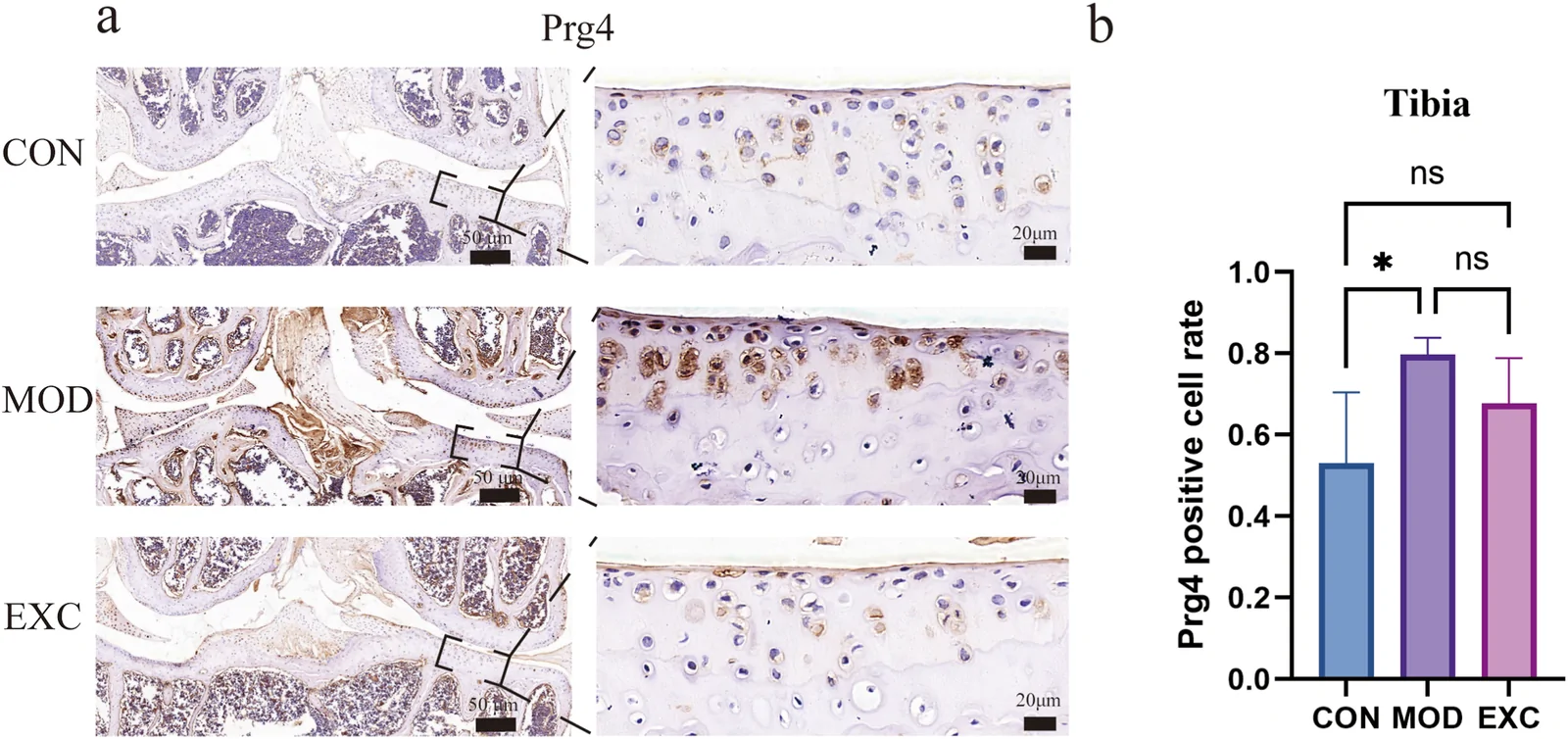
(a)The representative image of Prg4 immunohistochemistry, which reveals the higher express in moderate exercise, and the lower express in excessive exercise. (b) Quantification of tibial Prg4 positive cells. Data are expressed as the mean ± SD (n = 6 mice per group, *p < 0.05). Abbreviation: ns, no significance.

### 3.4 Moderate exercise attenuates cartilage degradation

X-ray imaging demonstrated no pathological abnormalities among the three groups [Fig. S4, Supplemental Information]. The balance between extracellular matrix (ECM) anabolic components (e.g., Col2a1, Aggrecan) and catabolic enzymes (e.g., MMPs, ADAMTS4/5) is critical for the progression of osteoarthritis [33]. Our results showed that moderate exercise (4-week, level treadmill running) did not significantly alter the expression levels of Col2a1 and Aggrecan in mouse knee joints [Fig. 6a and b]. Similarly, excessive exercise had no detectable effect on the expression of these two ECM anabolic markers.

**Fig. 6:**
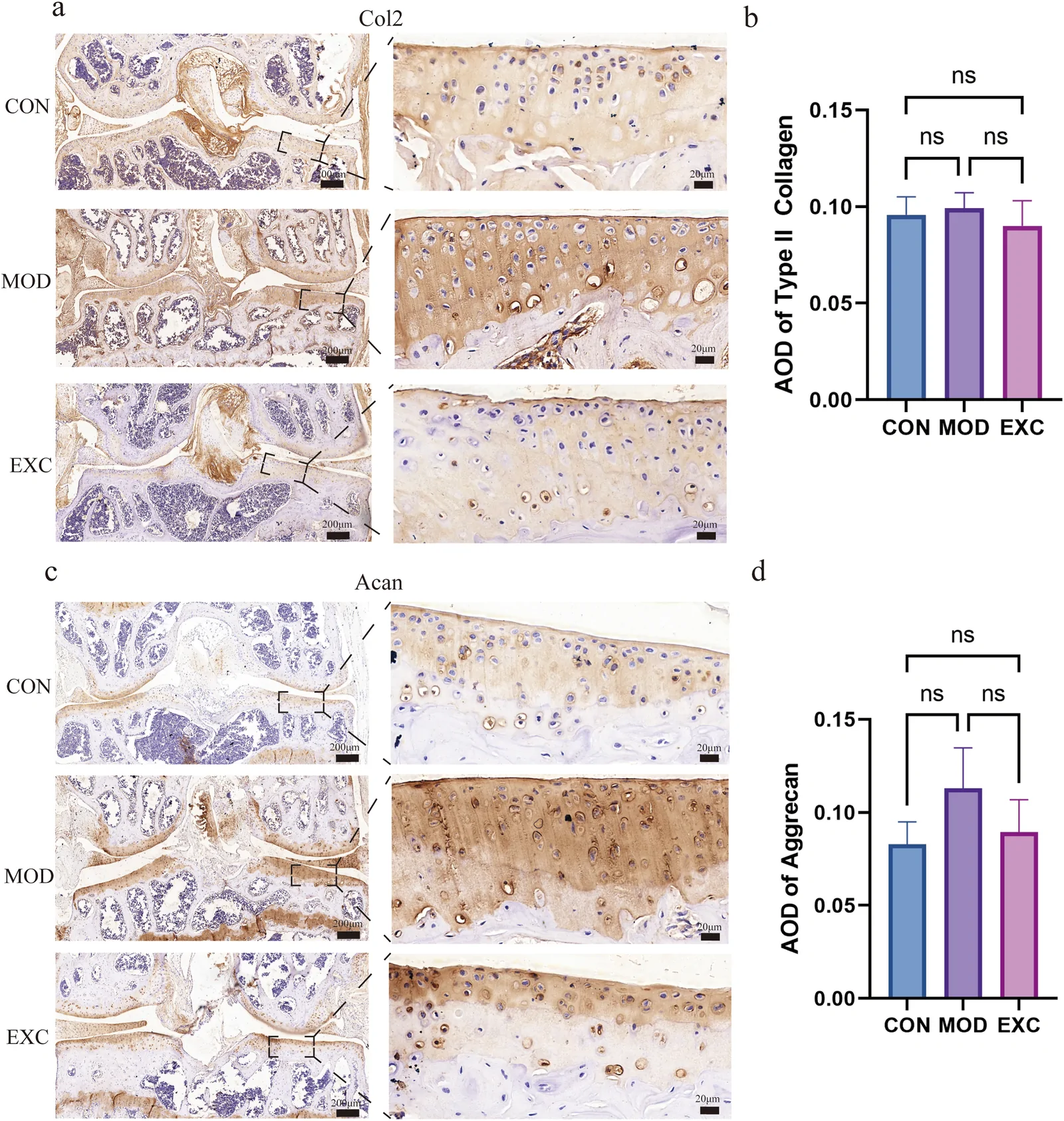
(a)The representative image of Collagen2 immunohistology in three groups. (b) Representative semi-quantitative analysis of collagen2. (c) The representative image of Collagen2 immunohistology in three groups. (d) Representative semi-quantitative analysis of Aggrecan. Data are expressed as the mean ± SD (n = 6 per group). Abbreviation: ns, no significance.

Collectively, these findings indicate that the exercise protocols used in the present study did not compromise the expression of cartilage anabolic markers.Unexpectedly, the percentage of Mmp13-positive cells showed no significant differences among the three groups [Fig. 7], the underlying mechanism of which warrants further exploration. A previous study reported that Caspase-3 levels correlated positively with lubricin expression [4]. Accordingly, we examined the expression pattern of Caspase-3 in mouse knee joints in response to treadmill exercise. Our results revealed that moderate exercise increased Caspase-3 activity, which declined with prolonged excessive loading, displaying an expression profile similar to that of Prg4 [Fig. 8]. Together, these results support a functional association between Caspase-3 and Prg4.

**Fig. 7:**
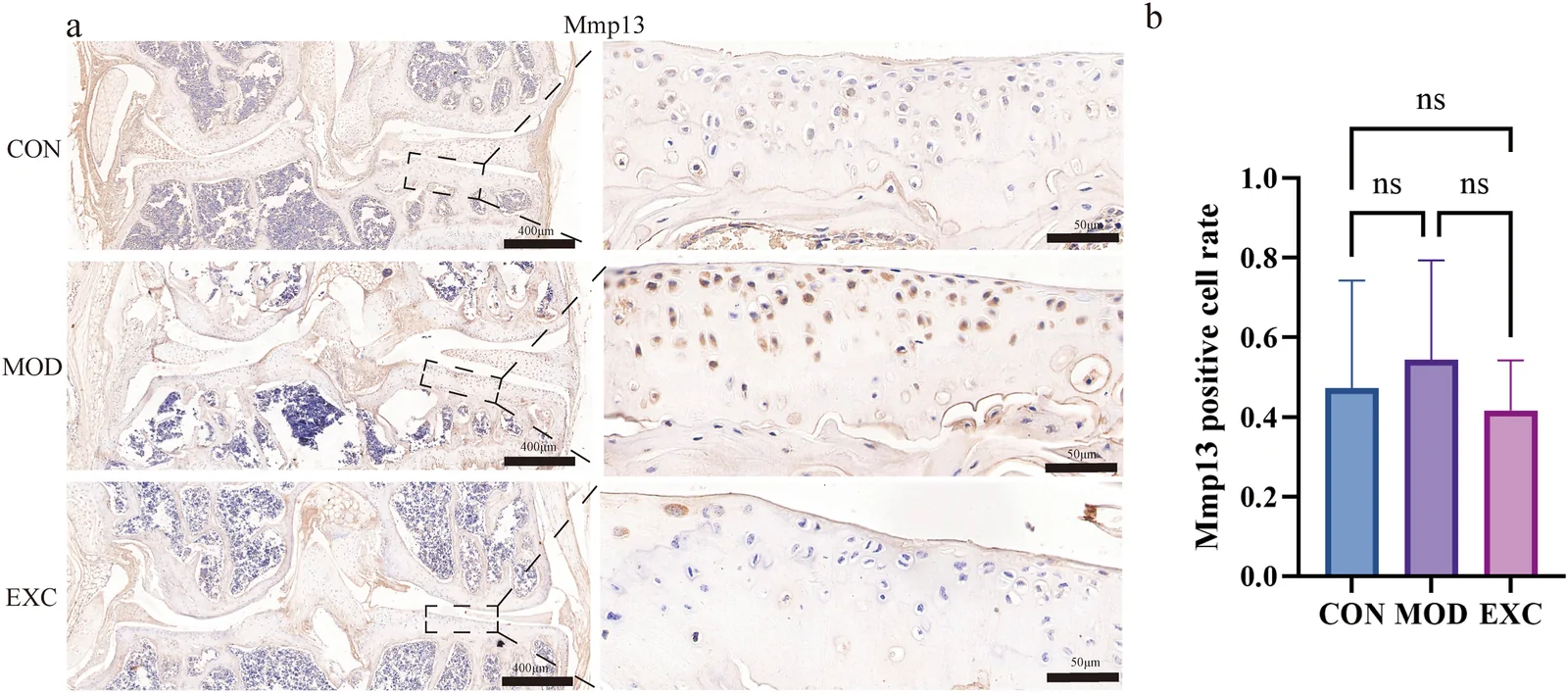
(a) The immunohistological images of Mmp13 in three groups. (b) Quantification of IHC staining of the number of positive cells of Mmp13. Data are expressed as the mean ± SD (n = 7 per group). Abbreviation: ns, no significance.

**Fig. 8:**
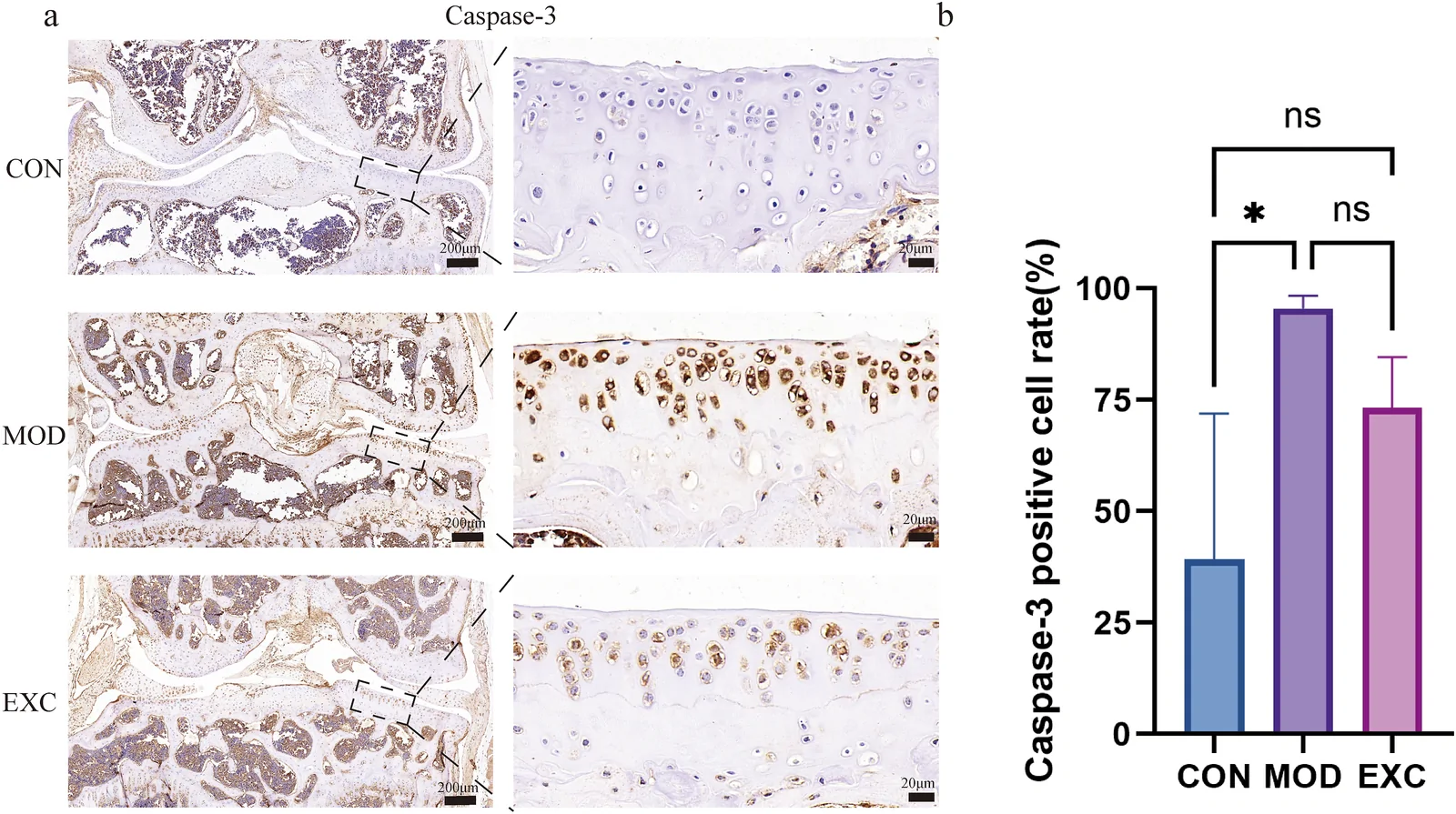
(a) The immunohistological images of Caspase-3 in three groups. (b) Quantification of IHC staining of the number of positive cells of Caspase-3. Data are expressed as the mean ± SD (n = 7 per group. *p < 0.05). Abbreviation: ns, no significance.

### 3.5 Progenitor cell and proliferation markers during exercise

To evaluate the effects of exercise on the pluripotency and proliferation of articular cartilage—particularly the superficial zone—we examined the expression of Cd34 [20] and Pcna [34] by immunohistochemical staining. No significant differences were observed between the control and moderate exercise groups. However, the number of CD34-positive cells in articular cartilage was significantly reduced in the excessive exercise group [Fig. 9], suggesting that intensive exercise regimens, especially overloading of the knee joint, may markedly suppress the expression of pluripotency markers in SFZ and cartilage cells. In line with this, staining for Pcna, a marker of cellular proliferation, showed no statistically significant differences among groups [Fig. 10], indicating that chondrocyte proliferative capacity was largely unaffected under the current exercise conditions. Further examination using additional stem cell markers such as CD105 and Sox2 [20] is warranted to fully characterize the dynamics of cartilage progenitor cells under different exercise modalities.

**Fig. 9:**
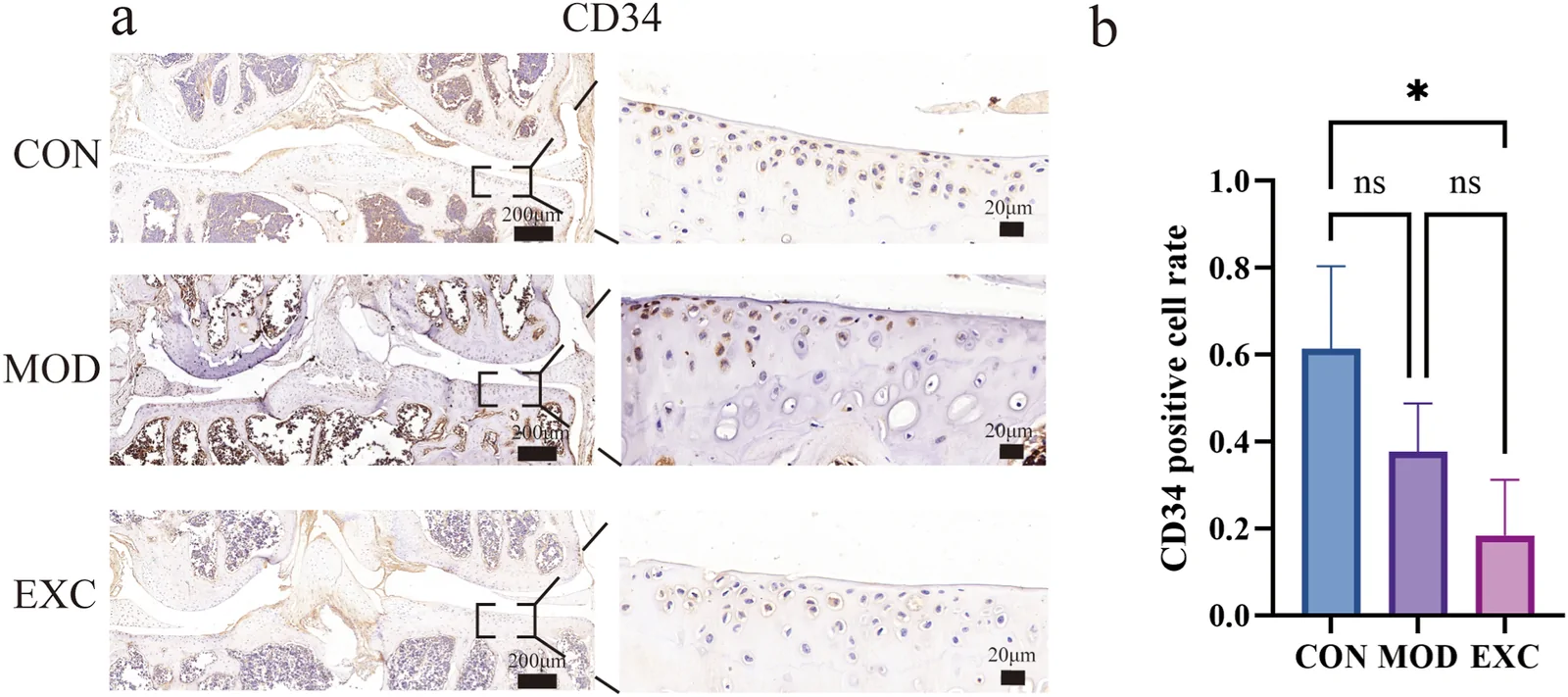
(a) The representative immunohistology image of CD34 in three groups. (b) Quantification of IHC staining of the number of positive cells of CD34. Data are expressed as the mean ± SD (n = 7 per group. *p < 0.05). Abbreviation: ns, no significance.

**Fig. 10:**
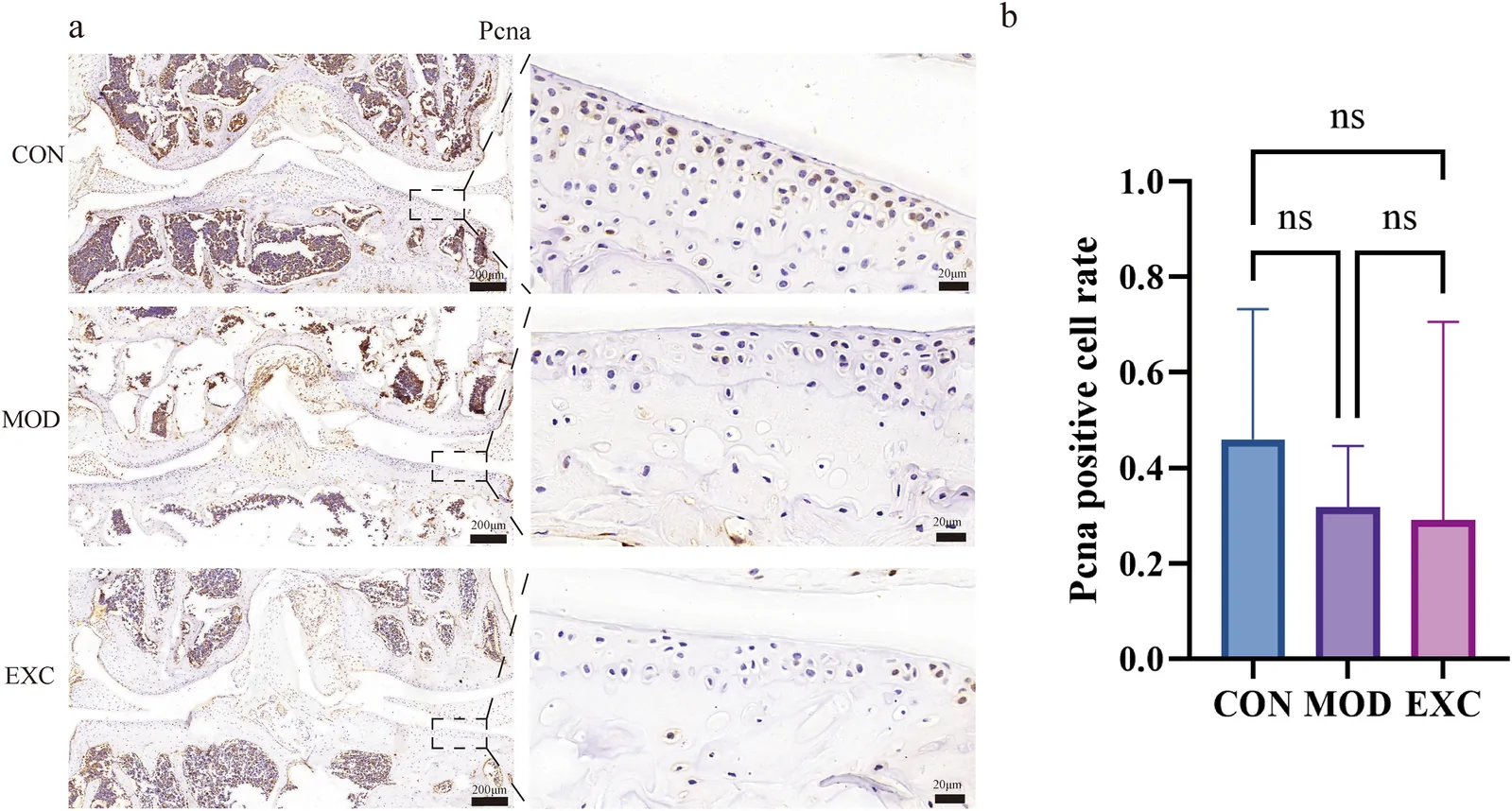
(a) The representative immunohistology image of Pcna in three groups. (b) Quantification of IHC staining of the number of positive cells of Pcna. Data are expressed as the mean ± SD (n = 6 per group). Abbreviation: ns, no significance.

## 4 Discussion

As the primary protective barrier of articular cartilage, the superficial zone (SFZ) plays an indispensable role in maintaining cartilage integrity. Our results demonstrate that moderate exercise enhances PRG4 expression, whereas excessive exercise diminishes this beneficial effect. Furthermore, Prg4-associated markers related to cellular proliferation, pluripotency, and extracellular matrix anabolism and catabolism each exhibited distinct expression patterns under different loading conditions. These findings may inform improved strategies for the early diagnosis and targeted treatment of osteoarthritis, with a particular focus on the superficial zone. Additionally, we propose that personalized exercise loading tailored to individual conditions may represent an optimal approach to preserving joint health.

To further explore the role of Prg4—which has been implicated in promoting cartilage growth [35]—we also analyzed the expression of CD34. As a key stemness marker, CD34 showed a downward trend in the excessive exercise group, indicative of impaired stemness in articular cartilage. For other matrix-related markers, including Collagen II, Aggrecan, and Mmp13, no significant differences were observed among the three groups. These molecules may only undergo marked alterations under more severe cartilage injury conditions, such as in ACLT or DMM models.

Furthermore, a previous study used μCT and histological analysis to quantify changes in articular cartilage thickness and volume following treadmill running [36]. Huesa et al. [27] demonstrated that moderate exercise effectively mitigated DMM-induced osteoarthritis in mice, as evidenced by both μCT and histological outcomes. In addition, Yin et al. [37] reported that moderate exercise significantly delayed the degeneration of superficial zone chondrocytes in a rat model of age-related OA. They further revealed that these effects were mediated via the TGFβ-pSmad2/3 signaling pathway, which translates mechanical loading into biological signals and modulates Prg4 expression. Conversely, excessive mechanical loading has been linked to the development of knee osteoarthritis in rats [38]. Collectively, these findings underscore that exercise modality and intensity are critical for maintaining synovial joint homeostasis. Excessive joint loading may trigger premature onset and progression of osteoarthritis.

In addition to the role of PRG4 in the repair and maintenance of injured and osteoarthritic articular cartilage, previous studies have reported that *Gdf5* is involved in joint remodeling and post-injury repair. *Gdf5* has also been linked to OA susceptibility and the specification of chondrogenic progenitor cells [39]. Another study further demonstrated that *Gdf5* serves as an early marker for joint interzone formation, preceding the expression of Prg4 during joint development [40].On the other hand, loss of Prg4 has been shown to induce elevated expression of Mmp9 and aberrant differentiation of SFZ cells, thereby contributing to early-onset OA and disrupted articular cartilage homeostasis [6]. Using Prg4-deficient mice, Anand et al. [41] observed distinct alterations in femoral size and shape compared with wild-type controls: distal femora in Prg4^−/−^ mice were enlarged, elongated anteroposteriorly, and narrowed mediolaterally, with the most prominent morphological changes detected in the trochlear groove, epicondyles, and medial condyle. These findings firmly establish that Prg4 is indispensable for joint homeostasis and morphological integrity. Consistent with these reports, our current study further supports the essential role of Prg4 in protecting articular cartilage and preserving the structural and functional stability of the superficial zone.

Regarding the apoptotic factor Caspase-3, which exhibited significantly higher activity in the moderate exercise group, it may be functionally associated with Prg4 expression. A previous study demonstrated that poly (ADP-ribose) polymerase-1 (PARP-1) knockout (KO) mice displayed more severe OA cartilage pathology and accelerated cartilage degeneration via the apoptotic pathway following DMM surgery. Furthermore, inhibition of PARP promoted apoptotic cell death in primary cultured chondrocytes, which was associated with increased cleaved Caspase-3 levels [42]. Another study further confirmed that the cleavage of caspases is a critical step in the apoptotic cascade [43]. Collectively, these findings suggest that the apoptotic pathway involved in our study and its association with exercise-induced changes require further investigation to clarify the underlying regulatory mechanisms.

Furthermore, Elena Kozhemyakina et al. [35]found the Prg4^GFPCreERT2^ is expressed by superficial articular chondrocytes in young mice, but expands into deeper regions of the articular cartilage as the animal age. Above all, they confirmed that the Prg4 extended into deeper cells from superficial cells as the mice age. In addition, CD34^*hi*^ subset of synovial fibroblasts potentially suppress synovitis and protect articular cartilage, and the regulation of these synovial cell subsets may lead to novel strategies for OA therapeutics [44]. To investigate whether the SFZ cells have stemness, we choose the CD34 [20] for its trait of cell stem marker. The outcomes showed that there is no significant differences between the experimental groups. Indeed, CD34 is reported as a stem marker for fibroblast lineage cells across multiorgans [45]. As a result, other stem cell markers should be further investigate.

Prg4-CreERT2; R26-tdTomato reporter mice represent a powerful tool for tracking the proliferation and migration of Prg4-positive cells in vivo [35]. Using the same lineage-tracing system, Tachibana et al. [46] identified a Rspo2-expressing subset within the Prg4+ cell population that plays a critical role in maintaining tendon and ligament homeostasis under pathological conditions.In the present study, we employed these reporter mice to characterize the spatiotemporal dynamics of Prg4+ cells in articular cartilage under varying exercise loads. Notably, similar Cre-dependent reporter models have also been applied to investigations of bone repair. He et al. [47] used Prg4-CreER; tdTomato mice and demonstrated that Prg4 is expressed in fibroadipogenic progenitors (FAPs), which contribute to acute muscle injury repair and fracture callus formation. In addition, Ninkovic et al. [24] reported that mesenchymal progenitor-derived proteoglycan 4 regulates chondrocyte-to-osteoblast transdifferentiation during fracture healing.

Future work will focus on Prg4-related signaling pathways, as well as the effects of a high-fat diet and long-term exercise on synovial fluid metabolomics and joint structural phenotypes in mice. These molecular profiles may serve as predictive biomarkers for disease severity and could be further applied to elucidate the mechanisms underlying obesity-associated knee osteoarthritis [48].In addition, previous studies have shown that SFZ cells cultured on chondrocyte-derived decellularized matrices exhibit enhanced proliferative and redifferentiation capacity [49]. We aim to explore the translational potential of this system in cartilage regeneration, especially with respect to the regulatory effects of physiological mechanical loading and gait patterns in vivo.

## 5 Conclusion

Our results highlight the critical role of Prg4 in response to exercise. With increasing physiological loading, Prg4 expression was significantly elevated and expanded from the superficial zone toward deeper cartilage regions. Under these conditions, Prg4 levels increased in a load-dependent manner, but this upregulation was not sustained under excessive, overburdening exercise.To further explore whether Prg4-positive cells possess stem cell-like properties, we examined CD34-positive cells and observed a downward trend in the excessive exercise group, revealing a distinct expression pattern during mechanical loading. The apoptotic pathway may be activated in our model, and the apoptotic effector Caspase-3 appears to be intrinsically linked to Prg4 expression and function.

## 6 Limitations

This study has several limitations. First, it lacks in vitro cellular experiments, such as the isolation and culture of SFZ cells to further characterize their phenotypic and functional differences under varied loading conditions. The molecular mechanisms governing Prg4 activation and its downstream signaling pathways also remain to be fully elucidated.In addition, the histological boundary between the superficial zone and middle zone could be more clearly defined and visualized to improve quantification accuracy. Moreover, validation of protein expression levels in knee joint tissues across experimental groups would strengthen the present findings. Finally, the exercise protocols used in this study could be further optimized and refined in future investigations.

## Informed consent statement

Not applicable.

## Author contributions

Conceptualization, C.W. and X.W.; methodology, P.T. and L.G.; validation, P.T., Y.S.; formal analysis, J.C., J.Z.; investigation, P.T.; resources, P.L., X.Z., C.W., and C.W.; data curation, P.T.; writing—original draft preparation, P.T., Y.S.; writing—review and editing, L.G., C.W., and X.W.; visualization, P.T.; project administration, L.G.; funding acquisition, X.W. and P.L. All authors have read and agreed to the published version of the manuscript.

## Institutional review board statement

Not applicable

## Data availability statement

The original contributions presented in the study are included in the article; further inquiries can be directed to the corresponding authors.

## Funding

This work was supported by the Regional Innovation Joint Fund of the 10.13039/501100001809National Natural Science Foundation of China (Integrated Project), Grant No. U23A6009 to X.W., the Central Leading Local Science and Technology Development Funds Project, Grant No. YDZJSX2024D065 to X.Z.

## Declaration of competing interest

The authors declare no conflicts of interest.

## References

[1] Liu J, Jia S, Yang Y, Piao L, Wang Z, Jin Z (2023). Exercise induced meteorin-like protects chondrocytes against inflammation and pyroptosis in osteoarthritis by inhibiting PI3K/Akt/NF-κB and NLRP3/caspase-1/GSDMD signaling. Biomed Pharmacother.

[2] da Silva LA, Thirupathi A, Colares MC, Haupenthal D, Venturini LM, Corrêa M (2023). The effectiveness of treadmill and swimming exercise in an animal model of osteoarthritis. Front Physiol.

[3] Morais GP, Chemerka C, Masson A, Seerattan RA, da Rocha AL, Krawetz R (2021). Excessive downhill training leads to early onset of knee osteoarthritis. Osteoarthr Cartil.

[4] Larson KM, Zhang L, Badger GJ, Jay GD. (2017). Early genetic restoration of lubricin expression in transgenic mice mitigates chondrocyte peroxynitrite release and caspase-3 activation. Osteoarthr Cartil.

[5] Yang DS, Dickerson EE, Zhang LX, Richendrfer H, Karamchedu PN, Badger GJ (2022). Quadruped gait and regulation of apoptotic factors in tibiofemoral joints following intra-articular rhPRG4 injection in Prg4 null mice. Int J Mol Sci.

[6] Maenohara Y, Chijimatsu R, Tachibana N, Uehara K, Xuan F, Mori D (2021). Lubricin contributes to homeostasis of articular cartilage by modulating differentiation of superficial zone cells. J Bone Miner Res.

[7] Ignatyeva N, Gavrilov N, Timashev PS, Medvedeva EV. (2024). Prg4-Expressing chondroprogenitor cells in the superficial zone of articular cartilage. Int J Mol Sci.

[8] Saito T. (2022). The superficial zone of articular cartilage. Inflamm Regen.

[9] Grad S, Gogolewski S, Alini M, Wimmer MA. (2006). Effects of simple and complex motion patterns on gene expression of chondrocytes seeded in 3D scaffolds. Tissue Eng.

[10] Rees SG, Davies JR, Tudor D, Flannery CR, Hughes CE, Dent CM (2002). Immunolocalisation and expression of proteoglycan 4 (cartilage superficial zone proteoglycan) in tendon. Matrix Biol.

[11] Marcelino J, Carpten JD, Suwairi WM, Gutierrez OM, Schwartz S, Robbins C (1999). CACP, encoding a secreted proteoglycan, is mutated in camptodactyly-arthropathy-coxa vara-pericarditis syndrome. Nat Genet.

[12] Xuan F, Yano F, Mori D, Chijimatsu R, Maenohara Y, Nakamoto H (2019). Wnt/β-catenin signaling contributes to articular cartilage homeostasis through lubricin induction in the superficial zone. Arthritis Res Ther.

[13] De Luca P, Kouroupis D, Viganò M, Perucca-Orfei C, Kaplan L, Zagra L (2019). Human diseased articular cartilage contains a mesenchymal stem cell-like population of chondroprogenitors with strong immunomodulatory responses. J Clin Med.

[14] Waller KA, Chin KE, Jay GD, Zhang LX, Teeple E, McAllister S (2017). Intra-articular recombinant human proteoglycan 4 mitigates cartilage damage after destabilization of the medial meniscus in the Yucatan minipig. Am J Sports Med.

[15] Ruan MZ, Erez A, Guse K, Dawson B, Bertin T, Chen Y (2013). Proteoglycan 4 expression protects against the development of osteoarthritis. Sci Transl Med.

[16] Ruan MZ, Cerullo V, Cela R, Clarke C, Lundgren-Akerlund E, Barry MA (2016). Treatment of osteoarthritis using a helper-dependent adenoviral vector retargeted to chondrocytes. Mol Ther Methods Clin Dev.

[17] Fu PJ, Zheng SY, Luo Y, Ren ZQ, Li ZH, Wang YP (2025). Prg4 and osteoarthritis: functions, regulatory factors, and treatment strategies. Biomedicines.

[18] Li L, Newton PT, Bouderlique T, Sejnohova M, Zikmund T, Kozhemyakina E (2017). Superficial cells are self-renewing chondrocyte progenitors, which form the articular cartilage in juvenile mice. Faseb j.

[19] Decker RS, Um HB, Dyment NA, Cottingham N, Usami Y, Enomoto-Iwamoto M (2017). Cell origin, volume and arrangement are drivers of articular cartilage formation, morphogenesis and response to injury in mouse limbs. Dev Biol.

[20] Yasuhara R, Ohta Y, Yuasa T, Kondo N, Hoang T, Addya S, Enomoto-Iwamoto M. (2011). Roles of β-catenin signaling in phenotypic expression and proliferation of articular cartilage superficial zone cells. Lab Invest.

[21] Roelofs AJ, Kania K, Rafipay AJ, Sambale M, Kuwahara ST, Collins FL (2020). Identification of the skeletal progenitor cells forming osteophytes in osteoarthritis. Ann Rheum Dis.

[22] Pineault KM, Song JY, Kozloff KM, Lucas D, Wellik DM. (2019). Hox11 expressing regional skeletal stem cells are progenitors for osteoblasts, chondrocytes and adipocytes throughout life. Nat Commun.

[23] Chavez RD, Sohn P, Serra R. (2019). Prg4 prevents osteoarthritis induced by dominant-negative interference of TGF-ß signaling in mice. PLoS One.

[24] Ninkovic N, Corpuz JM, Stahl A, Olsen A, Unger CM, Ardebili AA (2025). Mesenchymal progenitor-derived proteoglycan 4 regulates the transdifferentiation of chondrocytes into osteoblasts during fracture healing. Stem Cells Transl Med.

[25] Massengale M, Massengale JL, Benson CR, Baryawno N, Oki T, Steinhauser ML (2023). Adult Prg4+ progenitors repair long-term articular cartilage wounds in vivo. JCI Insight.

[26] Morais GP, da Rocha AL, Neave LM, de ALG, Leonard TR, Carvalho A (2020). Chronic uphill and downhill exercise protocols do not lead to sarcomerogenesis in mouse skeletal muscle. J Biomech.

[27] Huesa C, Dunning L, MacDougall K, Fegen M, Ortiz A, McCulloch K (2022). Moderate exercise protects against joint disease in a murine model of osteoarthritis. Front Physiol.

[28] Huang J, Xu Y, Li Y, Pang Y, Ding X, Zhou R (2025). HDAC4 regulates apoptosis in Acan-Cre(ERT2);HDAC4(d) (/d) mice with osteoarthritis by downregulating ATF4. FEBS Open Bio.

[29] Wu Y, Shen S, Chen J, Ni W, Wang Q, Zhou H (2023). Metabolite asymmetric dimethylarginine (ADMA) functions as a destabilization enhancer of SOX9 mediated by DDAH1 in osteoarthritis. Sci Adv.

[30] Yang P, Xiao Y, Chen L, Yang C, Cheng Q, Li H (2024). Targeting Fascin1 maintains chondrocytes phenotype and attenuates osteoarthritis development. Bone Res.

[31] Glasson SS, Chambers MG, Van Den Berg WB, Little CB. (2010). The OARSI histopathology initiative - recommendations for histological assessments of osteoarthritis in the mouse. Osteoarthr Cartil.

[32] Li J, Gui T, Yao L, Guo H, Lin YL, Lu J (2024). Synovium and infrapatellar fat pad share common mesenchymal progenitors and undergo coordinated changes in osteoarthritis. J Bone Miner Res.

[33] Deng Y, Lu J, Li W, Wu A, Zhang X, Tong W (2018). Reciprocal inhibition of YAP/TAZ and NF-κB regulates osteoarthritic cartilage degradation. Nat Commun.

[34] Habumugisha J, Okuda R, Hirose K, Kuwahara M, Wang Z, Ono M (2025). Critical requirement of senescence-associated CCN3 expression in CD44-Positive stem cells for osteoarthritis progression. Int J Mol Sci.

[35] Kozhemyakina E, Zhang M, Ionescu A, Ayturk UM, Ono N, Kobayashi A (2015). Identification of a Prg4-expressing articular cartilage progenitor cell population in mice. Arthritis Rheumatol.

[36] Kotwal N, Li J, Sandy J, Plaas A, Sumner DR. (2012). Initial application of EPIC-μCT to assess mouse articular cartilage morphology and composition: effects of aging and treadmill running. Osteoarthr Cartil.

[37] Yin Y, Zhang Y, Guo L, Li P, Wang D, Huang L (2023). Effect of moderate exercise on the superficial zone of articular cartilage in age-related osteoarthritis. Diagnostics.

[38] Zhou Z, Li Y, Xi K, Wu Y, Jia Y, Si Y (2026). Gubi Zhitong formula alleviates knee osteoarthritis induced by excessive mechanical loading in rats via regulating Piezo1 mediated Ca(2+) signaling pathway and phosphoinositide 3-Kinase/protein kinase B/nuclear factor kappa-B axis. J Ethnopharmacol.

[39] Kania K, Colella F, Riemen AHK, Wang H, Howard KA, Aigner T (2020). Regulation of Gdf5 expression in joint remodelling, repair and osteoarthritis. Sci Rep.

[40] Rhee DK, Marcelino J, Baker M, Gong Y, Smits P, Lefebvre V (2005). The secreted glycoprotein lubricin protects cartilage surfaces and inhibits synovial cell overgrowth. J Clin Investig.

[41] Masson AO, Devine J, Das N, Coveney CR, Hallgrimsson B, Liu Z (2025). Femur shape changes in Prg4-Deficient mice: morphological insights into joint well-being. Faseb j.

[42] Kim M, Ghosh M, Heo Y, Shin M, Min Y, Kim J (2026). PARP-1 prevents osteoarthritis pathogenesis by inhibiting apoptosis in chondrocytes: an animal study. Arthritis Res Ther.

[43] Sairanen T, Szepesi R, Karjalainen-Lindsberg ML, Saksi J, Paetau A, Lindsberg PJ. (2009). Neuronal caspase-3 and PARP-1 correlate differentially with apoptosis and necrosis in ischemic human stroke. Acta Neuropathol.

[44] Miyahara J, Omata Y, Chijimatsu R, Okada H, Ishikura H, Higuchi J (2025). CD34hi subset of synovial fibroblasts contributes to fibrotic phenotype of human knee osteoarthritis. JCI Insight.

[45] Buechler MB, Pradhan RN, Krishnamurty AT, Cox C, Calviello AK, Wang AW (2021). Cross-tissue organization of the fibroblast lineage. Nature.

[46] Tachibana N, Chijimatsu R, Okada H, Oichi T, Taniguchi Y, Maenohara Y (2022). RSPO2 defines a distinct undifferentiated progenitor in the tendon/ligament and suppresses ectopic ossification. Sci Adv.

[47] He Q, Lu J, Liang Q, Yao L, Sun T, Wang H (2025). Prg4+ fibroadipogenic progenitors in muscle are crucial for bone fracture repair. Proc Natl Acad Sci U S A.

[48] Hahn AK, Batushansky A, Rawle RA, Prado Lopes EB, June RK, Griffin TM. (2021). Effects of long-term exercise and a high-fat diet on synovial fluid metabolomics and joint structural phenotypes in mice: an integrated network analysis. Osteoarthr Cartil.

[49] Manzoni TJ, Ho A, Smull L, West VC, Waters JDV, Lemus K (2025). Enhanced superficial Zone chondrocyte expansion and redifferentiation by culture on chondrocyte-derived decellularized matrices. Cartilage.

