# Supplementary material for: Moderate exercise mobilizes Prg4+ cells in the superficial zone of articular cartilage and maintains cartilage homeostasis
Source: Biomed J. 2026 May 5;49(6):100989. doi: 10.1016/j.bj.2026.100989 (PMC13769563; doi:10.1016/j.bj.2026.100989)
Supplement: Supplementary file 1 [file mmc1.docx]

**Supplementary Materials:**

Table1

| Primary antibody | Company | Dilution | Information |
| --- | --- | --- | --- |
| CD34 | Zenbio, Chengdu,China | 1:100 | R380824,  monoclonal |
| Pcna | BOSTER, Beijing,China | 1:100 | BM3888,  monoclonal |
| Collagen2 | Wanleibio, Shenyang,China | 1:100 | WL03082,  polyclonal |
| Aggrecan | Proteintech, Wuhan,China | 1:100 | 13880-1-AP,  polyclonal |
| Mmp13 | ABclonal, Wuhan,ChinaMD | 1:100 | A11755,  polyclonal |
| Prg4 | Affinity,  Jiangsu,China | 1:100 | DF13331,  polyclonal |
| Caspase-3 | Beyotime,  Shanghai,China | 1:100 | AF0081,  polyclonal |

S1: The synovial of tracing mice in three group

S2: The meniscus of tracing mice in three group

S3: The synovial of WT mice in three group

S4: The representative X-ray images of control group, moderate exercise group and excessive moderate group.
